# The TimeStudio Project: An open source scientific workflow system for the behavioral and brain sciences

**DOI:** 10.3758/s13428-015-0616-x

**Published:** 2015-07-14

**Authors:** Pär Nyström, Terje Falck-Ytter, Gustaf Gredebäck

**Affiliations:** Uppsala Child & Babylab, Department of Psychology, Uppsala University, Box 1225, 751 42 Uppsala, Sweden; Pediatric Neuropsychiatry Unit Center of Neurodevelopmental Disorders at Karolinska Institutet (KIND) and Department of Women’s & Children’s Health, Child and Adolescent Psychiatry Research Center, Karolinska Institutet, Stockholm, Sweden

**Keywords:** Scientific workflow system, Open source, MATLAB, Time series, Eyetracking, Motion capture, Pupillometry

## Abstract

This article describes a new open source scientific workflow system, the TimeStudio Project, dedicated to the behavioral and brain sciences. The program is written in MATLAB and features a graphical user interface for the dynamic pipelining of computer algorithms developed as TimeStudio plugins. TimeStudio includes both a set of general plugins (for reading data files, modifying data structures, visualizing data structures, etc.) and a set of plugins specifically developed for the analysis of event-related eyetracking data as a proof of concept. It is possible to create custom plugins to integrate new or existing MATLAB code anywhere in a workflow, making TimeStudio a flexible workbench for organizing and performing a wide range of analyses. The system also features an integrated sharing and archiving tool for TimeStudio workflows, which can be used to share workflows both during the data analysis phase and after scientific publication. TimeStudio thus facilitates the reproduction and replication of scientific studies, increases the transparency of analyses, and reduces individual researchers’ analysis workload. The project website (http://timestudioproject.com) contains the latest releases of TimeStudio, together with documentation and user forums.

In April 2012, *Science* magazine published a policy forum article stating that custom-made analysis programs are “black boxes” in the scientific work flow (Morin et al., 2012). The article summarizes recent claims, from researchers (Barnes, 2010) and editors (Hanson, Sugden, & Alberts, 2011), stating that new tools are needed to expand the reporting and reproduction of data (Mesirov, 2010). Today, very few research groups make their analysis tools freely available (Morin et al., 2012).

Lack of transparency and detailed reporting may occur at many stages of research. Here we focus specifically on the black boxes related to data analysis—that is, how raw data are transformed to the summary statistics representing the “findings” of a study. This is important, since opaque analyses hinder scientific replications. The replication of research is a cornerstone of science (Noble, 2012), and individual studies need to be backed up by supporting results from other studies (Asendorpf et al., 2013; Ioannidis, 2005). However, replicability requires reproducibility (Asendorpf et al., 2013), and the analysis procedure should consequently be described in detail. The most detailed description is the actual source code that was used during an analysis, and researchers have been encouraged to publish both computer code and data in order to make the whole analysis accessible to others (Barnes, 2010; Mesirov, 2010; Peng, 2011).

Unfortunately it is not sufficient to supply only source code and data. Attempts to reproduce the results of published code and data are discouraging: only two of 54 results could be reproduced in Dewald, Thursby, and Anderson (1986); only 14 of 62 in McCullough, McGeary, and Harrison (2006); and only nine of 117 in McCullough, McGeary, and Harrison (2008). The main obstacles were often related to different platform behaviors (Windows, Mac, Linux, etc.) and missing dependencies (external programming libraries, third-party software, etc.). Thus, simple posting of the source code and data is not enough. In order for the reproduction process to work more smoothly, the code and data also need to be prepared for cross-platform reproduction in a way that is appropriate for each individual research field (Curcin & Ghanem, 2008; McCullough et al., 2008). Unfortunately, there is currently no “gold standard” for meeting this goal (Curcin & Ghanem, 2008).

Although transparency and replicability may be perceived as abstract concepts, they affect individual researchers in concrete ways. The negative consequences of poor transparency and replicability typically involve unnecessary time spent on trouble shooting programs and the development of functionally identical programs. The positive consequences include the increased impact of research findings: Piwowar, Day, and Fridsma (2007) reported a correlation between shared detailed research data and increased literature impact, with up to 69 % increased citations. Sharing research data and analyses is apparently beneficial not only for the general scientific community, but also for individual researchers.

Two general goals for science are thus to (1) increase transparency and (2) increase replicability. Two practical problems with meeting these goals are that (1) source code and data are not made available to the desired extent, and (2) when they are, the material may not be prepared for use by others. In this article, we introduce the TimeStudio Project, a fully transparent system dedicated to the analysis, reproduction, and sharing of quantitative data in order to address both the practical problems and general scientific goals. In the Method section, we present an overview of the project. In the Results section, we present a case study illustrating the most important concepts of TimeStudio and show how analyses can be shared and reproduced. In the Discussion section, we address how TimeStudio use may impact the research community, how the software relates to similar systems, and how the project will be developed in the future.

## Method

### Overview

The framework presented here, named “the TimeStudio Project,” is a novel open source scientific workflow system. Scientific workflow systems are designed to automatize the execution of a set of algorithms that operate on data resources in a scientific analysis (Deelman, Gannon, Shields, & Taylor, 2009).

The TimeStudio software consists of three parts: core software, plugins, and Web resources. The core software is developed and maintained by the Uppsala Child- and Babylab as open source and includes the functionality to organize an analysis into a workflow. The core also handles the sharing of workflows. The TimeStudio plugins are accessible from the core program but are conceptually separated from the core, since researchers may develop custom plugins in their own fashion. Finally, the Web resources include the online TimeStudio database and project Web pages with user manuals, installation instructions, and a user forum for support.

**Fig. 1:**
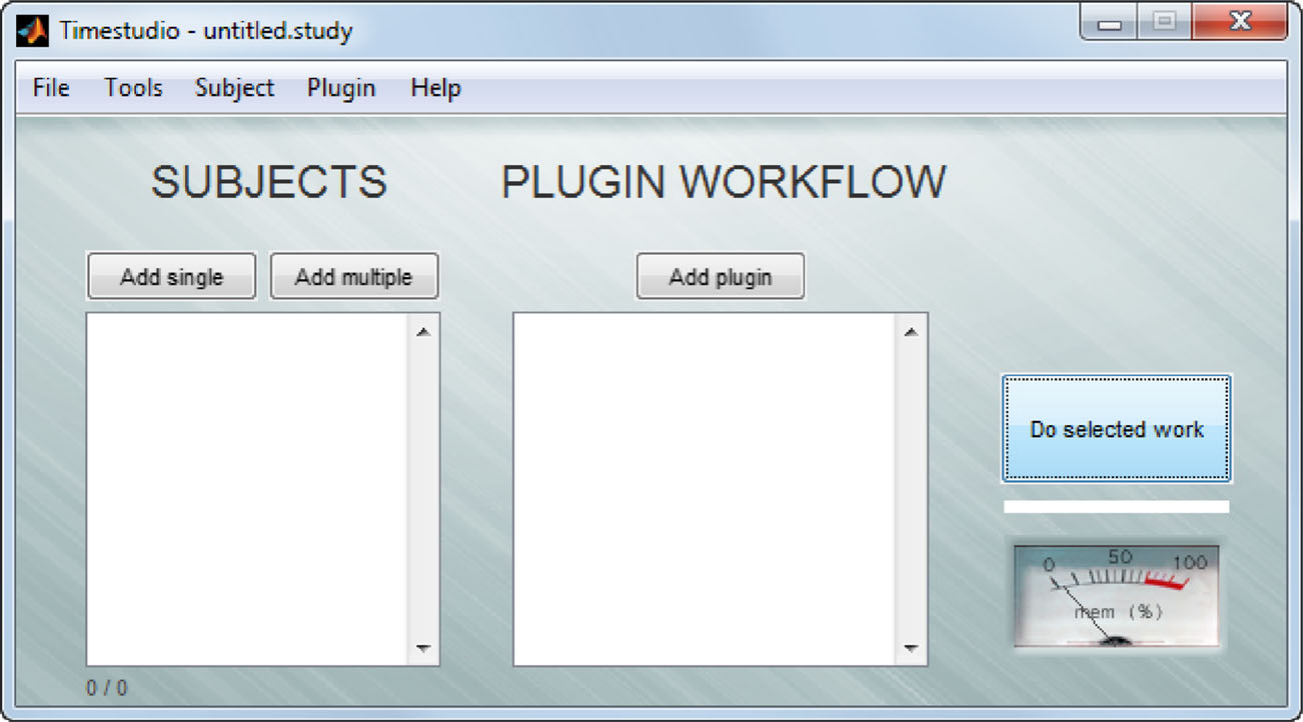
Main window of TimeStudio. The system uses terminology and structures stemming from the behavioral sciences. Data are stored within one or several “subjects” that are accessible from the list box on the left. The data are then processed by plugins that are arranged in a workflow that is defined in the list box on the right. The workflow is executed by pressing the button labeled “Do selected work”

In practice, the TimeStudio core software allows researchers to arrange a sequence of TimeStudio plugins within a graphical user interface (GUI) (Fig. 1), in order to organize an analysis into a step-by-step protocol. Each plugin contains computer algorithms to perform a specific task, such as reading data files, filtering time series data, or visualizing data. The plugins are typically used to process data structures grouped into “subjects” (Fig. 1, “Subjects” list), bearing on the terminology from traditional experimental design within the behavioral and brain sciences, where the same subject may contain data from multiple recording sessions and/or multiple measures. The Subjects list and the plugin sequence together constitute a TimeStudio workflow. By arranging a TimeStudio workflow, the whole analysis becomes prepared for sharing and reproduction, including both data files (attached to the subject’s data structures) and computer algorithms (stored within TimeStudio plugins). TimeStudio offers functionality to facilitate the publishing of workflows to the TimeStudio database from the main GUI. After sharing a workflow, the whole analysis can be reproduced by a single button click on another computer running TimeStudio. Thus, TimeStudio addresses the two practical problems mentioned in the introduction, by (1) preparing an analysis to be used by others, and (2) offering a solution to share workflows effortlessly. Through these means the reproduction and replication of studies is facilitated, and by using open source software the analytic process becomes fully transparent. In this article, TimeStudio will be illustrated using time series data derived from the behavioral sciences (as reflected in the terminology and structure with “Subjects,” etc.). However, the general approach is applicable to all fields of science and is not restricted to time series analysis.

The TimeStudio core and plugins require the MATLAB (MATLAB Release 2012b, The MathWorks, Inc., Natick, MA, USA) environment, release 2012b or later. TimeStudio is mainly developed and tested on computers running Windows, with secondary platform-specific testing and debugging on Mac OS X and Linux. Its visual appearance differs slightly between operating systems. The latest version of the TimeStudio software and the most updated information about the TimeStudio Project can be found on the project’s Web pages: http://timestudioproject.com. TimeStudio versions are also available at FigShare (Nyström, 2015). The Web resources also include installation instructions, documentation, user forums, and a custom database for storing TimeStudio workflows.

### TimeStudio plugins

All data processing is done within the plugins and is controlled through the core software. The available plugins define which analyses are possible within TimeStudio without additional plugin development. During the installation of TimeStudio, a set of general plugins are supplied, which allows for basic file reading and time series processing. In addition to these generic plugins that allow the analysis of time series data, a set of plugins for analyzing eyetracking data in novel ways are also supplied (and are also used in a case study below). For other types of analyses, it is possible to create new custom plugins within the MATLAB environment.

All TimeStudio plugins have separate GUIs that may contain any number of textboxes, checkboxes, lists, etc. The parameters of these user components are stored as plugin settings. This is useful if the same plugin should be used with different parameters (e.g., a low-pass filter could be used in many contexts, but with different frequency cutoffs). For those who are not trained in programming, it is thus possible to use the included plugins and to adjust the available parameters from the GUIs. However, all computer code is accessible from within the GUIs to allow full transparency. Since MATLAB is a high-level programming language that is widely used within academia, the actual algorithms will be readable by many users. The TimeStudio plugins are standard MATLAB function files that contain three mandatory function calls. The mandatory function calls will ensure that all TimeStudio plugins have a standardized GUI and that the plugin will be compatible with other instances of TimeStudio and future releases.

### Workflows and sharing

**Fig. 2:**
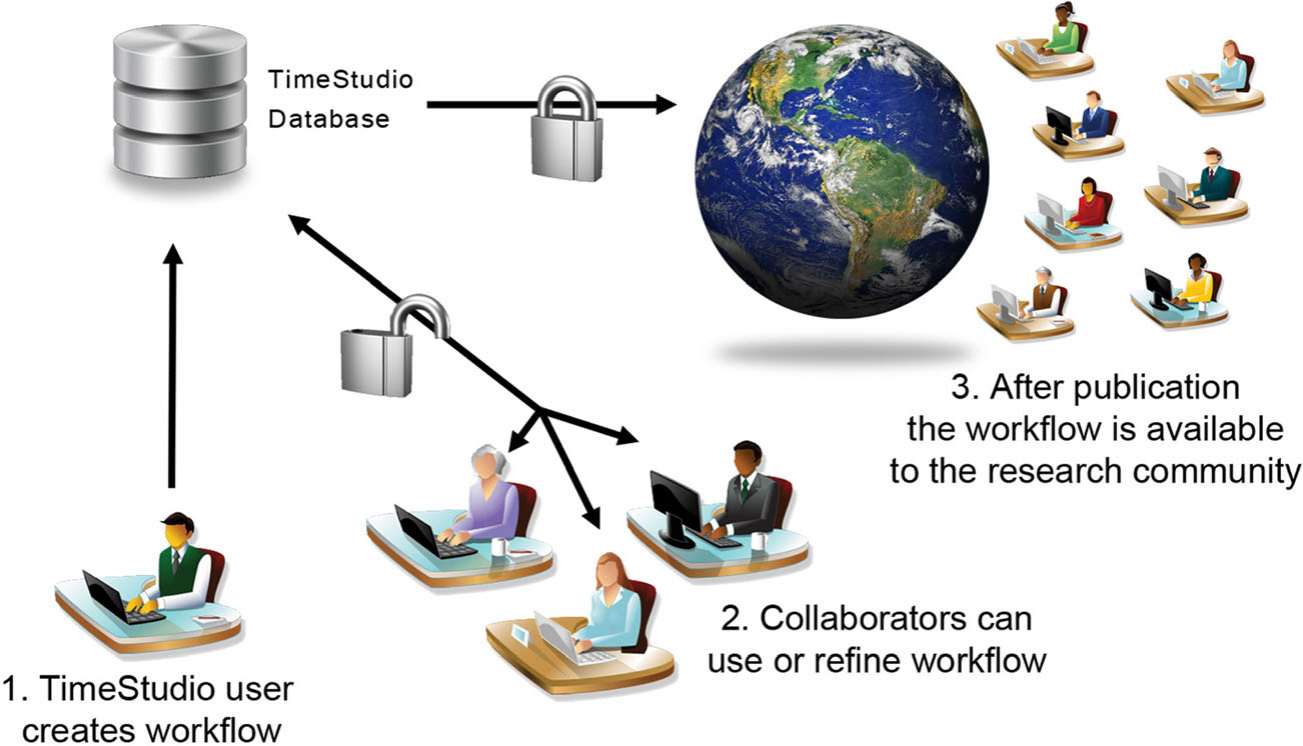
Example of workflow development and sharing in three steps

A TimeStudio analysis arranges a sequence of plugins in a workflow. The workflow also contains all parameter settings for each plugin, and when the workflow is run, the subject data are processed according to the plugin sequence and the plugin settings. The plugin order settings are therefore important to the final outcome. During the design of an analysis, TimeStudio makes it possible to adjust the workflow and share it with other team members. A GUI functionality facilitates such collaboration on workflows both within labs and between labs. During workflow development, the analysis can be uploaded to the TimeStudio database in an unlocked state. Collaborators can then download and make appropriate adjustments before uploading the new version. However, after publication the TimeStudio study should be locked so that the study can be securely archived and available to the research community (Fig. 2). Each archived workflow is given a unique workflow identification code (UWID), which can be cited in scientific papers to facilitate transparency and reuse.

**Fig. 3:**
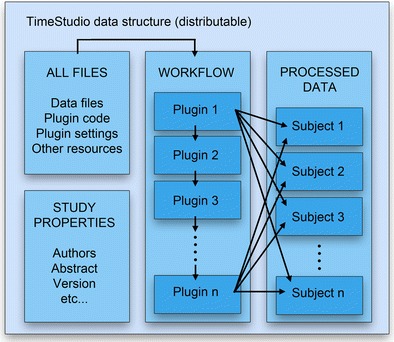
Example of a TimeStudio data structure. All original files are contained within the structure and can be extracted to the hard drive. The workflow consists of a list of plugins with algorithms that are applied to the subject data. Each subject may have an arbitrary number of substructures (usually containing data from different methodologies)

The TimeStudio framework avoids reproducibility problems stemming from missing dependencies and platform-specific features by constraining the computational environment to MATLAB and by using a single data structure (Fig. 3). This data structure, called a “TimeStudio study,” will store the full analysis: subject data, workflow of algorithms, plugin source code, and the necessary dependencies. In this way, the analysis can be reproduced by a single buttonpress from any other user running TimeStudio.

To illustrate the points made above, a concrete example of how TimeStudio and TimeStudio UWIDs can be used in practice is presented in the Results section below.

### Ethics statement

The case study conformed to Standard 8 of the American Psychological Association’s Ethical Principles of Psychologist and Code of Conduct.

## Results

### Case study

The case study presented here is intended as a demonstration of TimeStudio and an example of how workflows can be shared and reproduced within the TimeStudio framework. This case study will not provide a step-by-step description of how the analysis was constructed; such information can be found within the documentation of TimeStudio at http://timestudioproject.com/manualspage.

The case study shows a novel analysis workflow that extracts event-related pupil responses during mathematical task solving of different degrees of difficulty. The stimuli were designed to resemble a study published in *Science* by Ahern and Beatty (1979) as closely as possible: Two numbers were presented after each other, and the subjects should silently multiply them. The stimuli started with an empty white background for 2 s. A black text multiplicand was presented 2 s after stimulus onset, and the multiplier was presented after 4 s. The numbers pairs were selected from the ranges 1 to 9 (*easy*), 6 to 14 (*medium*), or 11 to 19 (*difficult*). The gaze position, pupil size, and stimulus event triggers were recorded using a Tobii 1750 eyetracker and exported to text files (Tobii Clear View analysis software: combined data, with gaze data and event data in the same file). The original study showed that approximately 6–8 s after stimulus onset, the pupil was more dilated during tasks that demanded higher cognitive load, and this finding is replicated here.

The case study analysis can be reproduced on computers running MATLAB (r2012b or later) and requires that TimeStudio be installed. Updated installation instructions can be found on the project website, http://timestudioproject.com/getting-started, but in most cases it is enough to enter the following command in the MATLAB command window:

 >> eval(urlread('http://timestudioproject.com/install.php'));

During installation, the user is prompted to specify a folder where TimeStudio should be installed. By pressing the “Enter” key, the default folder “timestudio” will be created in the current working directory. Downloading the core system and the workflow may take some time, depending on the Internet connection, but after finishing the installation TimeStudio can be started, and the main window should appear (as in Fig. 1, but with empty list boxes).

The actual workflow is archived in the TimeStudio database (UWID = ts-6b6-27c) and is downloaded by using the TimeStudio “File” menu alternative “Open uwid.” After downloading, the workflow consists of five plugins:

1. Core_read_file: a flexible text file reader that reads text files in which the data are arranged in rows and columns. By setting the parameters in the plugin GUI, it is possible to link the text file columns to different data fields in the “subjects” data structure. Core_read_file can also be used to create events from column data for later event-related analyses. In this example, the plugin parsed the eyetracker text files so that gaze position, pupil size, and the event data for stimulus onsets were extracted for each subject.
2. Events_modification: a plugin that can modify events. In this example, the previous plugin extracted events that marked the onset of every stimulus presentation. However, the events were not categorized into different conditions. In this example, the event names were modified to group the stimuli into the conditions “easy,” “medium,” and “difficult.”
3. Core_interpolate_gaps: a plugin that can interpolate intervals with missing data. The plugin can replace NaN values (“Not a Number,” MATLAB terminology) with the nearest neighbor or through linear or cubic interpolation. Options are also available to exclude interpolation if the inserted data do not meet specific criteria (such as by exceeding a specific range or a jump in the data points). In the case study analysis, gaps smaller than five samples were replaced through a linear interpolation, since eyetracking data may include short gaps during which the eyetracker could not get a reliable gaze estimate.
4. Core_filter_moving_average: a plugin that performs a sliding-window average (or median) filter on a time series. In the case study, the plugin performed a moving median filter with a window length of five samples in both directions (equaling an 11-sample window with no phase shift), in order to remove spurious outlier pupil sizes. Also, a moving-average filter with the same window length was applied in order to smooth the pupil time series.
5. Core_event_related_data_extraction: a plugin that extracts time segments (trials) from a time series in relation to events. Prior to data extraction, the time series data can be modified according to the specific demands of the analysis. For example, the segments can be baseline corrected, and several measures can be calculated for a given time interval within the segment: average value, median value, min value, max value, latency to min value, latency to max value, and range of values. In this example, trials starting at the events “easy,” “medium,” and “difficult” were extracted and baseline corrected between 0 and 2 s after the start of each trial. When running this plugin, two outputs are created. First, the average time series for the three conditions were visualized together with confidence intervals (Fig. 4). Second, the average pupil sizes for individual subjects and trials were extracted between 6 and 7 s after the start of each trial and output in a text table.

**Fig. 4:**
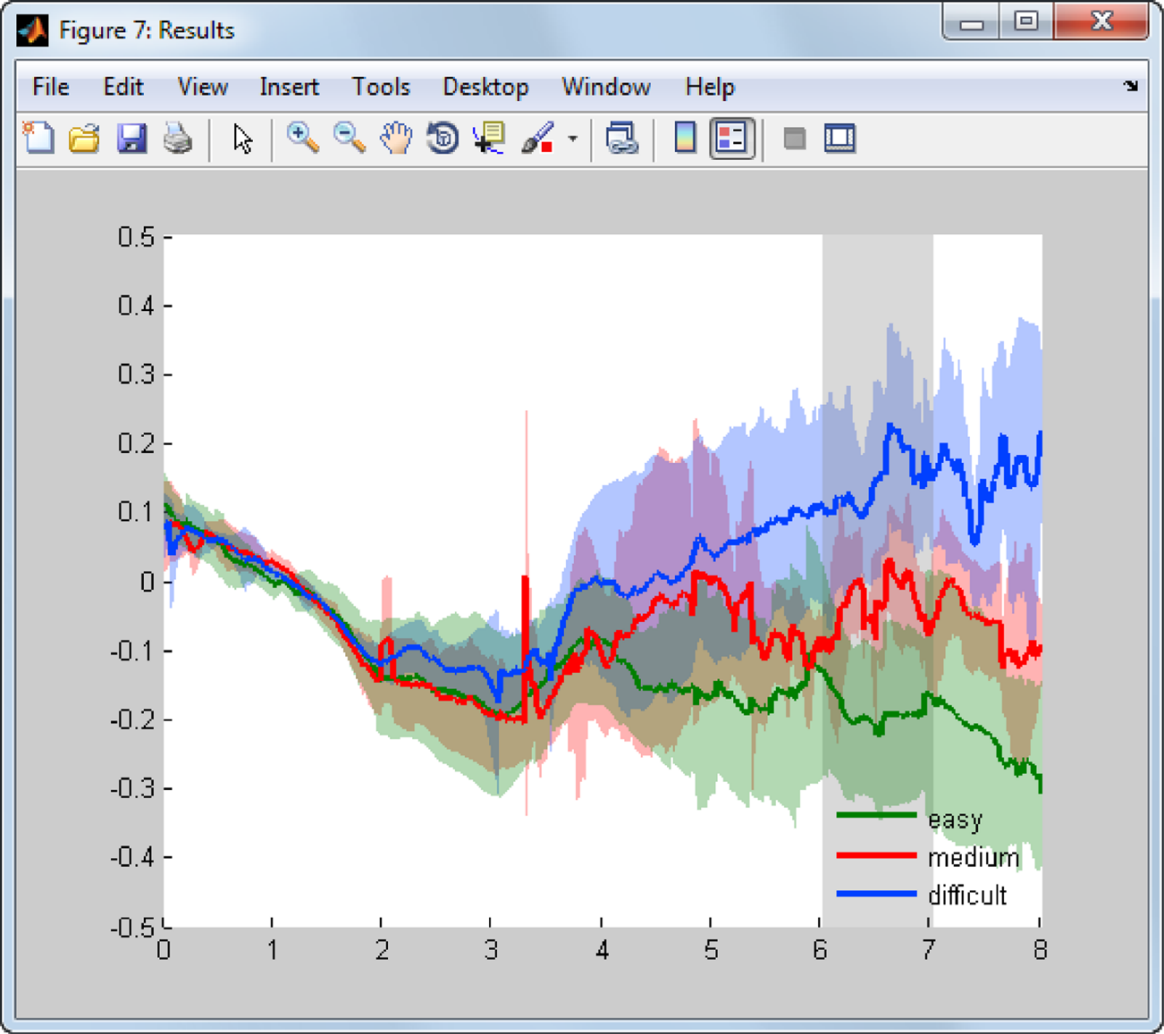
Graph showing average pupil size per condition
6. The text output was copied and pasted into statistical software (SPSS, in our case) for further testing.

To start running the workflow, the button “Do selected work” should be pressed. Processing time may vary depending on the local computer performance. A progress bar in the main window indicates how much of the workflow is processed.

One can click any plugin in the main window in order to inspect and modify the settings of the plugin. Users of TimeStudio may wish to change the settings within the plugins in order to know how specific parameters can alter the results of the workflow (e.g., in order to assess the stability of the results). If any of the plugin settings have been modified, it is necessary to process the workflow again to update the output results. In order to rerun the workflow, select the subjects and plugins you want to use and press the button labeled “Do selected work.”

### How to add custom plugins to a workflow

One advantage of general scientific workflow systems over more specialized analysis tools is the possibility to pipeline all analysis steps required for publication, including preprocessing, visualization, and statistical tests. Although the core plugins that are currently available in TimeStudio do not include statistical tests, TimeStudio can be extended with custom plugins to perform most types of computations. In this section, we demonstrate how the workflow in the above case study can be extended with a custom plugin to perform a simple bootstrap statistical test and to display summary data as a line plot. In order to fully understand the steps below, it is highly recommended that you download and run the case study above and follow these steps in practice.

1. First of all, a plugin .m file should be created. This can be done by using the “Create plugin” alternative in the main window’s “Plugin” menu. A dialog will pop up and prompt for a plugin type (in this example, we use “test”) and a plugin name (in this example, we use “bootstrap”). There is also a drop-down menu with plugin templates that have different amounts of example code; for this plugin, the template “template_basic_example_group.m” should be selected. Click the “Create plugin now” button. When the dialog closes, TimeStudio will automatically create a new .m file called “test_bootstrap.m” and open it in the MATLAB editor.
2. The MATLAB code for the plugin can now be written into the .m file. This particular plugin template includes code that sets up the setting window, the format of the help section, basic operations that communicate with the TimeStudio core, and a few example user interfaces (two text boxes, a dropdown menu, and a check box). Also, text comments show where code for the setting window and for the plugin processing should go. Insert the following code directly after the comment “% Your code for plugin processing should start here” (currently at line 57 in the “test_bootstrap.m” file), and save the changes (Ctrl + S on Windows systems).

% loop through subjects

for s = 1:numel(TS.selected)

% get data from individual subjects

values = TS.ALLSUBJ(TS.selected(s)).eyetracking.eventrelateddata;

% calculate mean value for easy trials

data(1, s) = nanmean ([values.easy.trialvalues]);

% calculate mean value for medium trials

data(2, s) = nanmean([values. medium . trialvalues]);

% calculate mean value for difficult trials

data(3, s) = nanmean([values . difficult . trialvalues]);

end

% create figure

figure;

% plot individual mean values

plot(data, '.-', 'MarkerSize', 15);

% format axes

set(gca, 'XLim', [0 4], 'XTick', 1:3)

% add text to x axis

set(gca, 'XTickLabel', {'easy', 'medium', 'difficult'});

% add a label for y axis

ylabel('Pupil size');

% calculate bootstrapped p-value

pvalue = TSbootstrap(data(1,:), data(3,:), 5000);

% add title to graph

title(['Bootstrapped p-value between easy and difficult: ' …

num2str(pvalue, '%0.3f')]);

3. In order to add the new plugin to the workflow, click the “Add plugin” button in the main window. A context menu will appear, from which the “test_bootstrap” plugin can be selected (under the “custom” and “test” submenus). Once the plugin is selected, the setting window will appear, containing the example user controls defined in the “test_bootstrap.m” file. Since we do not use any of these controls, click “Use plugin” to close the setting window and add the plugin to the workflow. You may be prompted to save the setting with a different name than “default”; in this case, save as “noName” and press “Use plugin” again.
4. To make TimeStudio run the new plugin, select only the “test_bootstrap” plugin from the workflow list in the main window. Select all subjects in the Subjects list and press the button “Do selected work” to run the plugin. TimeStudio will now start processing and display a figure with the scatterplot and the statistical test results in the title.

Developing new plugins requires basic knowledge of both the MATLAB programming language and the TimeStudio data structure. Also, knowing some of the TimeStudio wrapper functions for GUIs may also speed up the development of new plugins. This knowledge can be acquired from the project Web resources: from the manual for plugin development at http://timestudioproject.com/manualspage, from the TimeStudio user forum, and from the template plugins.

## Discussion

In the introduction, we posed two general goals for applied scientific research. These goals are to (1) increase transparency and (2) increase replicability. We also identified two practical problems with meeting these goals. These problems were (1) that source code and data are not made available to the desired extent, and (2) when they are, the materials may not be prepared for use by others. The TimeStudio Project is focused on solving the practical problems for individual researchers in order to achieve higher scientific goals.

Problem 1, that source code and data are not made available to the desired extent, is addressed by TimeStudio through an integrated publishing route to the TimeStudio database. This route requires minimal effort: A workflow can be uploaded with five mouse clicks and will be assigned a sharable UWID. Also, by including the UWID in a publication, it becomes easy for other researchers to download the analysis in order to replicate the study and continue on that line of research. Such spreading of paradigms is beneficial for the authors of the workflow and may encourage more researchers to publish their workflows (which include source code and data). Another feasible way of sharing would be to export a TimeStudio workflow to a binary file using the standard MATLAB .mat format. Such files can be transferred as any other file: by e-mail, by uploading to an FTP server, etc. The exported binary format also makes it possible to share TimeStudio workflows through available open access repositories (see the further discussion below).

Problem 2, that the materials may not be prepared for use by others, is addressed by TimeStudio by using a scientific workflow structure. By placing all computational code within TimeStudio plugins and ordering these plugins within a workflow, all required information for reproduction is automatically available and prepared for sharing. It is thus possible to say that using TimeStudio is the same as preparing the analysis for use by others.

Since TimeStudio prepares the materials, the system is also responsible for their ease of use. In TimeStudio we have designed the user interfaces to minimize the number of user interactions (such as mouse clicks) for the most common functions. For instance, once the workflow has been opened in TimeStudio, the analysis can be run by a single mouse click in the main GUI. All other functionality is available through a small set of user controls (Subjects list, Workflow list, GUI menus, etc.), which at the same time allow an overview of the most important features of the workflow.

TimeStudio thus offer solutions to the two practical problems outlined above. However, reaching the general scientific goals (increasing replicability and transparency) will require that more researchers actually use a scientific workflow system such as TimeStudio. What will motivate researchers to start using TimeStudio? We have identified at least three additional arguments that favor TimeStudio against time series analyses conducted using custom analysis tools created in-house or by using third-part analysis packages:

First of all, the project was initiated in order to make a wide variety of analysis tools available to the broader research community for free. Most scientists are not programmers (Barnes, 2010), suggesting that researchers are often restricted to the availability of analysis tools in standard analysis packages. TimeStudio offers a way to use plugins that have been developed by others within the same general framework. Since any researcher may program their own plugins or hire a programmer to develop additional plugins, the availability of analysis tools is controlled by the research community, rather than by third-party actors. Also, the general approach of TimeStudio allows for the integration of data from multiple measuring techniques, so there are no restrictions to particular research fields or measuring devices.

Second, the lack of structured training in programming and code documentation increases the risk that programing errors will impact the analysis of data (Morin et al., 2012). Posting one’s analysis tools and making them easy to reproduce under an open source license makes it easier to communicate how data are processed. An equally important effect will be a more rapid transfer of novel analysis methods to a wider community of researchers. This is beneficial for the inventors of novel methods and could counterbalance publication bias.

Third, recent advancements in open access publication (Laakso et al., 2011) and data storage requirements (National Institutes of Health, 2003; National Science Board, 2011) need to be followed up by the storage and publication of analysis tools (Morin et al., 2012). The integrated export and sharing functions in TimeStudio make it easy to meets such requirements.

A positive side effect of the properties of TimeStudio is that TimeStudio could help minimize the risk of scientific misconduct. It is becoming increasingly clear, both to researchers (Martinson, Anderson, & De Vries, 2005) and the wider community (The Economist, 2011; Wade, 2010) that research is afflicted by fraud (Ranstam, Ryd, & Önsten, 2000; Swazey, Anderson, & Louis, 1993) and scientific misconduct (Martinson et al., 2005). By making data and analysis tools transferable, more researchers can participate hands-on in analyzing data, something we believe will decrease analysis errors and reduce everyday scientific misconduct.

### TimeStudio and other alternatives

It is worth discussing how other scientific workflow systems relate to TimeStudio. Indeed, the concept of scientific workflows has been implemented in many other software suites (Deelman et al., 2009), such as Discovery Net (Rowe, Kalaitzopoulos, Osmond, Ghanem, & Guo, 2003), Pegasus (Deelman et al., 2005), Kepler (Altintas et al., 2004), Taverna (Wolstencroft et al., 2013), Triana (Taylor, Shields, Wang, & Harrison, 2007), and KNIME (Berthold et al., 2008), among others. Although this situation could raise a competition between systems, this will not necessarily be the case. In reality, it is unlikely that one system would outperform and replace all others (Curcin & Ghanem, 2008). Since different research fields have their own project architectures, it is in fact favorable to have a range of workflow systems that are optimized for various types of research projects (Curcin & Ghanem, 2008). The previously mentioned workflow systems have been developed with the focus of joining separate data archives or separate computational resources in order to perform large-scale data mining and grid computing (often in the fields of the life sciences, genome mapping, or astronomy; Curcin, Ghanem, Wendel, & Guo, 2007; Deelman et al., 2009; Taylor, Deelman, & Gannon, 2006). However, many researchers may not need very extensive or complex workflow systems. TimeStudio is geared toward users who need a lightweight workflow system that has few concepts to learn at the beginning but is dynamic enough to extend to a wide range of scientific applications. TimeStudio itself and TimeStudio plugin development are all kept within MATLAB, and if custom-developed plugins are necessary, there is no need to change environment. Because of the argumentation above, we regard TimeStudio to be a complementary alternative to other workflow systems, and not in competition with them. Considering the widespread use of MATLAB within academia and the vast amount of smaller-scale studies published every year that do not benefit from any scientific workflow system, we believe that TimeStudio could be an alternative that would suit many researchers in different research fields.

The use of MATLAB in research during the last decades has resulted in a broad range of available analysis tools. In psychology and neuroscience, popular toolkits such as the SPM, FieldTrip, and EEGLAB (Delorme & Makeig, 2004) suites are role models. Although it is possible to take advantage of such tools and utilize them from TimeStudio plugins, it is the responsibility of the individual researcher to make sure that the licenses of such external resources are not violated and that dependencies are included in the exported TimeStudio workflow. TimeStudio, in turn, is licensed using a liberal MIT license (http://figshare.com/licensing) that makes it possible to use algorithms and data structures in other frameworks. This makes TimeStudio resources fit into larger systems of data analysis, such as Comp-Engine Time Series (Fulcher, Little, & Jones, 2013), which aim at enhancing scientific quality across disciplines. However, adjustment of the TimeStudio export format may be required to conform to particular third-party systems.

It may be argued that using MATLAB as the core platform works against the ambition of making scientific analyses accessible, since MATLAB is proprietary software. On the other hand, MATLAB is the preferred platform for a substantial part of the researchers working with behavioral or neuroscience data, and it is available to employees at more than 5,000 universities and colleges (according to http://mathworks.com/academia/). It is therefore likely that MATLAB will remain a popular programming language for many years. Instead of excluding these researchers or expecting them to change programming environments, we hope that TimeStudio will build on and enhance their existing competences, and make their algorithms transparent and usable by others. Most open access alternatives to MATLAB (such as GNU Octave, R, or Python) have the capacity to open MATLAB files, and thereby exported TimeStudio studies. Although the actual plugin source code would have to be reimplemented to run in another programming environment, it is possible to retrieve all data files, the workflow of plugins, and the plugin settings, as well as the MATLAB source code. The most important point is that all resources are prepared for reproduction, which would be of great help if a whole analysis should be migrated to another programming language. We hope that other agile frameworks similar to TimeStudio will emerge for other popular programming languages, so that researchers can choose the most appropriate programming environment and still utilize the advantages of lightweight scientific workflow systems.

### Who should use TimeStudio, and why

As we have argued above, there are many benefits for the general research society in the use of scientific workflow systems. But what are the main reasons for individual researchers to start using TimeStudio?

First, we believe that many users will start using TimeStudio in order to open and explore an existing workflow, and not necessarily to create their own workflow. The possibility to use a GUI to examine workflows makes analyses available to anyone, and not only to those who can script their own workflows using MATLAB, R, Python, or other programming languages. Since TimeStudio workflows can be shared using short references (UWIDs or hyperlinks), and since the procedure to get a full analysis workflow running on a local computer is streamlined to be simple, the effort for first-time users is minimal. If the novel user wants to use parts of the existing workflow in his or her own research, this is most easily done by continuing to use TimeStudio. Thus, many researchers may start using TimeStudio because all analyses on this platform are easily accessible and prepared for reproduction. This will save effort by reusing existing plugins and workflows, and will make researchers less dependent on programming skills.

The experienced programmer, on the other hand, will need to devote less time to interface development and to instructing or supporting less experienced collaborators. By creating TimeStudio custom plugins, all code can automatically be delivered with familiar user interfaces and prepared for sharing. TimeStudio custom plugins may also appear less homemade than in-house scripts, which signifies that the developer is aware of the importance of accessibility and usability.

Finally, from a lab leader perspective it is important to make the communication and distribution of work tasks easy within the research group. TimeStudio can be used to organize an accessible and transparent infrastructure. For researchers working in larger networks, TimeStudio may facilitate the harmonization and quality control of shared experimental and analytic procedures. Thus, taken together, there are clear benefits of using TimeStudio for researchers ranging from research assistants to senior lab leaders.

### Future development

As a novel software suite, TimeStudio has many possible directions of development. First of all, the TimeStudio Project has already proved to be an efficient working tool within different labs (such as within our labs at Uppsala University, Sweden; at Karolinska Institute, Sweden; and externally at the University of Tampere, Finland), and we are committed to the maintenance and development of the TimeStudio core and new plugins for the coming years. Many analyses can be performed by combining the existing core plugins, but we want to point out that the core plugin library is likely to be extended as TimeStudio becomes used in more contexts. The core plugins should therefore not be regarded as static, but as a dynamic toolkit based on user needs. Importantly, we will continue to improve TimeStudio in light of the feedback from users. Such feedback can be posted both through the TimeStudio Web resources (the user forum and contact forms) and through the report functions integrated in the TimeStudio main window and settings windows. During the development of TimeStudio, we have used the system in a wide range of in-house applications, such as plugins for analyzing video, skin conductance, 3-D motion tracking, and functional near infrared spectroscopy. We have also used custom TimeStudio plugins to perform statistical tests, analyze longitudinal studies using scalar values from each time point, and integrate questionnaire tests scores with experimental data. We have found the current GUI to work well for all of these applications, and it is possible that these in-house plugins will be included as core plugins in later releases.

Another important area of development is to facilitate independent plugin development outside of our labs. In the future, we will invite researchers without programming knowledge to order tailor-made plugins and mediate between professional MATLAB software engineers, but now TimeStudio is already at a stage at which external researchers can contribute with new plugins. In the extension of the case study, a new plugin was created, and a similar cut-and-paste strategy can be used to integrate already-developed MATLAB code into TimeStudio plugins (or see the manual for plugin development at http://timestudioproject.com/manualspage). The TimeStudio user forum can be used to ask questions, share knowledge, and suggest improvements that will help custom plugin development.

The TimeStudio database is a limited resource that will need to be considered in the future. One option would be to enhance the existing database performance, which would require increased funding. Another, more likely option would be to transfer the archive functionality to a mature external solution for open access scientific data storage, such as Dryad (http://datadryad.org/).We also have ongoing discussions with the DiVA archive (DiVA, 2013; Müller, Klosa, Andersson, & Hansson, 2003) and FigShare (http://figshare.com). Although all of these options are viable, there will not be a final decision until the actual usage of TimeStudio increases. In the meanwhile, the benefits of using existing archiving solutions can already be utilized by exporting the TimeStudio studies for binary upload.

The fact that TimeStudio will continue to develop raises questions regarding versioning and backward compatibility. It is important to note that TimeStudio does not use or implement any versioning system for front-end users. This deliberate choice stems from experience with novice users that have failed to install or use the versioning systems correctly and from the aim to keep TimeStudio lightweight. Instead, all core software is archived in the TimeStudio Project Web resources and on FigShare (doi:10.6084/m9.figshare.1293476). All officially released versions of TimeStudio later than version 2.3 are available as separate installations, so that users may choose which version to use. In the same vein, rather than having a dedicated versioning system for plugins, different versions are treated as separate plugins that can be stored and developed using separate UWIDs or exported studies. An important feature is that TimeStudio stores plugins in a workflow-specific folder, so that when a workflow is reproduced it can use the correct plugin folders. Another advantage of this is that one can use TimeStudio .study files to bundle a set of plugins and safely share them between users, even though two or more plugins have the same name, similar to branching in versioning control systems.

Backward compatibility is related to versioning. TimeStudio uses a specific file structure for plugins and settings, and as long as this file structure is preserved, TimeStudio will scan and use the available plugins correctly. The TimeStudio studies also include information about which version of TimeStudio was used to run the workflow, and it is possible to include the core software in the .study file to ensure that an old workflow can be run. In this way, older studies can be run with older versions of TimeStudio if newer versions of TimeStudio are incompatible. However, considering the overall purpose of TimeStudio (to make scientific workflows as accessible as possible), backward compatibility would be of the highest priority during future development. We also encourage users to include as much relevant information as possible when reporting TimeStudio workflows in scientific journals, including: a citation to this article and the TimeStudio UWID, TimeStudio version, and MATLAB version.

### Summary

In summary, TimeStudio is a novel scientific workflow system that aims to increase transparency and replicability in research. At the same time, TimeStudio should facilitate the spreading of successful paradigms and increase efficiency when developing new scientific analyses. All use and development is kept within the MATLAB environment, which is widely used within academia. The most updated information about the project, together with documentation and user forums, is available on the project website: http://timestudioproject.com.
